# Harnessing Human Placental Membrane‐Derived Bioinks: Characterization and Applications in Bioprinting and Vasculogenesis

**DOI:** 10.1002/adhm.202303370

**Published:** 2023-11-20

**Authors:** Léo Comperat, Lise Chagot, Sarah Massot, Marie‐Laure Stachowicz, Nathalie Dusserre, Chantal Médina, Théo Desigaux, Jean‐William Dupuy, Jean‐Christophe Fricain, Hugo Oliveira

**Affiliations:** ^1^ University of Bordeaux Tissue Bioengineering U1026 Bordeaux F‐33076 France; ^2^ Inserm U1026 Tissue Bioengineering ART BioPrint Bordeaux F‐33076 France; ^3^ CHU Bordeaux Services d'Odontologie et de Santé Buccale Bordeaux F‐33076 France; ^4^ University of Bordeaux Plateforme Protéome Bordeaux 33000 France

**Keywords:** angiogenesis, bioink, bioprinting, extracellular matrix extraction, human amniotic membrane

## Abstract

Bioprinting applications in the clinical field generate great interest, but developing suitable biomaterial inks for medical settings is a challenge. Placental tissues offer a promising solution due to their abundance, stability, and status as medical waste. They contain basement membrane components, have a clinical history, and support angiogenesis. This study formulates bioinks from two placental tissues, amnion (AM) and chorion (CHO), and compares their unique extracellular matrix (ECM) and growth factor compositions. Rheological properties of the bioinks are evaluated for bioprinting and maturation of human endothelial cells. Both AM and Cho‐derived bioinks sustained human endothelial cell viability, proliferation, and maturation, promoting optimal vasculogenesis. These bioinks derived from human sources have significant potential for tissue engineering applications, particularly in supporting vasculogenesis. This research contributes to the advancement of tissue engineering and regenerative medicine, bringing everyone closer to clinically viable bioprinting solutions using placental tissues as valuable biomaterials.

## Graphical abstract

Bioprinting for clinical applications holds great promise but requires appropriate biomaterial inks. Placental tissues, often discarded as medical waste, offer a compelling solution due to their abundance, stability, established clinical history, and angiogenic properties. This study develops bioinks from amnion and chorion, investigating their extracellular matrix and growth factor compositions. These bioinks, fostering human endothelial cell viability and vasculogenesis, hold significant potential for tissue engineering, advancing the feasibility of clinically viable bioprinting using valuable placental biomaterials.

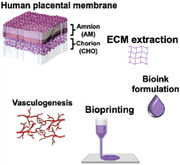

## 1 Introduction

In recent years, 3D bioprinting has garnered significant interest for its potential to create implantable tissues and for its ability to provide greater control over the 3D deposition of cells and biomaterials.^[^ ^1^ ^]^ However, and in spite of the great enthusiasm drawn into the field, proof‐of‐principle achievements have been limited to tissues with simple architecture, like skin and cardiac patches.^[^ ^2^, ^3^, ^4^, ^5^ ^]^ One of the main challenges in advancing this technology lies in the lack of suitable biomaterial inks that can support the optimal maturation of bioprinted cells.^[^ ^6^, ^7^ ^]^ Indeed, the design of biomaterial inks should not be based solely on printability or rheological properties, but also, and probably more importantly, it should consider their biological interaction and instructive capacity to the bioprinted cells. We, and others, have focused on the use of extracellular matrix (ECM)‐based matrices for the creation of physiological relevant biomaterial inks that have mainly relied on the use of xenogeneic ECM (e.g., porcine gelatin, rat or bovine type I collagen).^[^ ^8^, ^9^, ^10^, ^11^, ^12^ ^]^ Indeed, and in spite of their inherent capacity to replicate the ECM tissue composition, animal‐derived materials pose several concerns related to their xenotoxicity and immune response, upon human implantation.^[^ ^13^ ^]^ Decellularization, which removes most of the cellular content while preserving the ECM, has been shown to reduce immune response in the host. Nonetheless, xenogeneic decellularized ECM tissues have shown to induce severe inflammatory reactions, fibrosis and premature tissue degradation upon implantation in humans.^[^ ^14^, ^15^, ^16^ ^]^ Other challenges related to the use of xenograft materials can be identified, namely the potential interspecies disease transmission, informed consent related issues and animal welfare. As such, alternative ECM‐based material sources should be envisaged, particularly when aiming at the fabrication of human implantable tissues.

Placental tissues, specifically the human amniotic membrane (AM) and chorionic membrane (CHO), have been extensively studied for use in biomedical applications. The AM is the membrane that surrounds the fetus, while the CHO is adjacent to the maternal tissue. The AM has been used for therapeutic purposes since the beginning of the 20th century, particularly in dermatology, skin repair and ophthalmology,^[^ ^17^ ^]^ and profuse undergoing clinical trials may pave the road for further medical applications for this human sourced biomaterial. Placental membrane use is therefore still gaining traction, mainly due to its inherent biological properties, namely: able to promote wound healing, low immune response, antifibrotic, antimicrobial, rich source of growth factors, cytokines, interesting mechanical properties and naturally composed by ECM components (e.g., hyaluronic acid, collagens, laminin, fibronectin, and proteoglycans).^[^ ^17^, ^18^, ^19^ ^]^ As such, it represents an attractive source of human ECM that can find use of tissue engineering applications, particularly in bioprinting, as a human sourced biomaterial ink. Moreover, placental tissues are considered surgical waste that can be obtained after elective caesarean surgery and with donor consent. It is thus a highly abundant, readily available, and cost‐effective human biological tissue that poses little ethical concerns.

A broad number of studies have indeed used this human sourced biomaterial for a wide range of tissue engineering applications.^[^ ^20^ ^]^ Of particular interest, some of these studies have focused on the obtention of pepsin‐solubilized hydrogels, with or without subsequent chemical modifications that enabled photopolymerization, that can then be used for tissue engineering applications.^[^ ^21^, ^22^, ^23^, ^24^, ^25^ ^]^ Nonetheless these studies have focused on the AM alone and have not explored the application of such biomaterials for a direct bioprinting application.

In this study, we developed a method to isolate and decellularize both the AM and CHO membranes, tested two ECM extraction approaches (i.e., through pepsin digestion or acidic dissolution), introduce methacrylate groups to enable photopolymerization and improved printability by using hyaluronic acid (HyA). We aimed at establishing if the two different membranes (AM and CHO) could exert distinct biological responses and also compared them with one of the gold standards of animal sourced ECM, type I collagen (Coll).

The rationale behind our selection of AM (amniotic membrane) and CHO (chorionic membrane) is rooted in the extensive body of research that has already investigated these membranes for clinical applications. Additionally, the reported variations in their growth factor content^[^ ^26^ ^]^ have served as a motivating factor for us to independently investigate these two distinct ECM sources. As proof of principle we have focused on one of the basic aspects of regeneration for most tissues, vasculogenesis, and tested the creation of bioinks using AM or CHO, human umbilical vein endothelial cells (HUVECs) and human fibroblasts. In this work we could show that these novel biomaterial inks can sustain the bioprinting and maturation of human endothelial cells and demonstrate their potential for future bioprinting applications.

## 2 Results and Discussion

### 2.1 Extracellular Matrix Preparation, Extraction, and Characterization

Placental tissues, known for their bioactive properties and originating from human sources, hold great potential as biomaterials for biofabrication. The successful use of non‐viable placental tissue allografts in clinical settings indicates the therapeutic possibilities of using placental tissues as biomaterial inks, which is the primary focus of this study.

**Figure 1:**
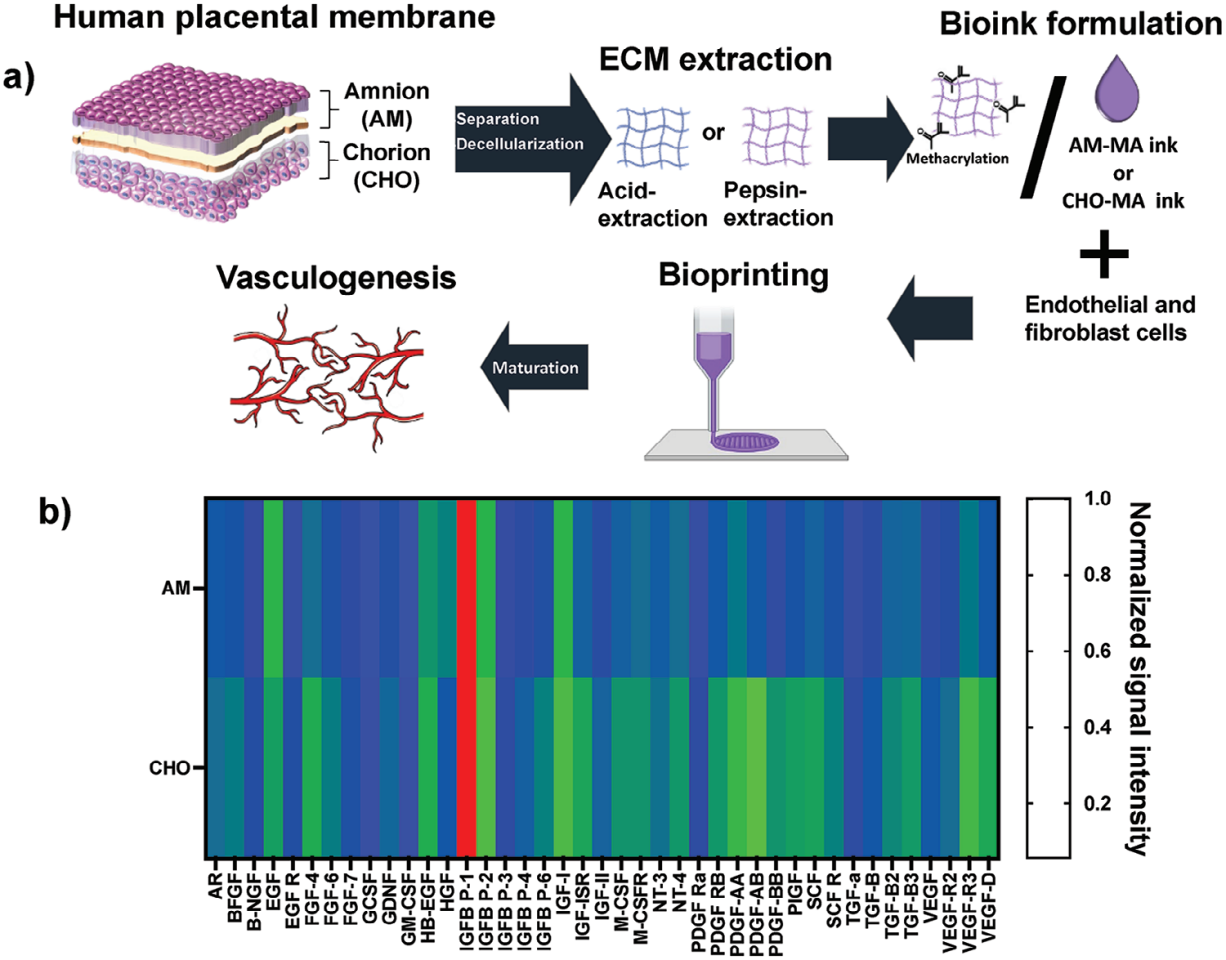
a) Schematic representation of the approach used to accomplish the creation of a human placental membrane‐derived biomaterial ink, able to be bioprinted and to support in vitro vasculogenesis. b) Heatmap analysis of 41 human growth factors present in decellularized amniotic membrane (AM) or decellularized chorion (CHO), expressed as normalized signal intensity. Average values compiled from 3 independent membrane donors (*n* = 3).

The overall procedure used to attain the preparation of a biomaterial ink, based on human placental membranes, is illustrated in **Figure** **1**a. First, to establish the impact of different placental membrane sheets on ink composition, physico‐chemical characteristics and biological output, we isolated and separated both the AM and CHO membranes and proceeded with their decellularization. Then, two established extracellular matrix extraction approaches, pepsin digestion or acidic extraction, were tested and compared. To attain photopolymerization and optimal viscosity, suitable for microextrusion bioprinting, we proceeded with methacrylation and association with methacrylated hyaluronic acid, based on previous reports.^[^ ^27^ ^]^ We then characterized these inks, validated its capacity to attain the bioprinting of human umbilical vein endothelial cells (HUVECs) in co‐culture with human skin fibroblasts (HSFs) and tested the capacity of the developed bioinks to support vasculogenesis.

The placental membrane's pro‐regenerative, anti‐inflammatory, and antibacterial properties are primarily attributed to the release of growth factors and cytokines, which align with the tissue's role during gestation.^[^ ^28^ ^]^

As such, to evaluate cytokine and growth factor content in the AM and CHO was key in terms of matrix characterization. We proceeded with the relative quantification of an array of 41 human growth factors. As observed in Figure 1b the totality of the growth factors tested could be detected in the used array. Also, the comparison of AM and CHO showed a significant increase for 23 human growth factors present in CHO, in relation to AM, 17 growth factors showed similar expression for both groups and 1 factor was lower for CHO in relation to AM (see Figures S1A,B, Supporting information).

**Figure 2:**
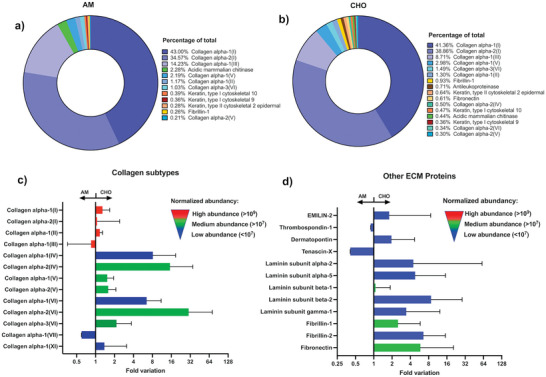
Abundance of the identified extracellular matrix proteins, expressed as percentage of total, for decellularized amnion (AM, a)) and chorion (CHO, b)). Normalized fold variation abundancy, expressed as fold variation of CHO versus AM, for collagen subtypes c) and other ECM derived proteins d) (Please note the code of color for high abundance (red) medium (green) and low abundance). Data representative of 3 independent AM and CHO membranes (Data expressed as median with interquartile range).

Mass spectrometry analysis was used to assess if different protein composition profiles could be established for decellularized AM and CHO. This relative analysis, using 3 independent AM and 3 independent CHO human samples, enabled to identify 1186 peptides, with correspondent 956 proteins. As observed in **Figure** **2**a,b, the relative abundance of major ECM‐derived components was not significantly different between the two groups and showed a major preponderance of collagen composition (collagen alpha I and alpha II). Additionally, fold variation of both AM and CHO were compared and the most relevant variations can be depicted in Figure 2c,d, Table S1A,B, Supporting Information). As expected, for the higher abundant components (red labeling) no major modulation could be observed. Nonetheless, for medium and lower abundant components (green and blue labeling, respectively) one can observe that in the great majority of matrisome‐associated proteins there is a relative increase for CHO, in relation to AM. Collagens I, III, IV, V, VI; laminin, fibrillin, and fibronectin are ECM components well‐established as crucial elements in both AM and CHO.^[^ ^29^ ^]^ Nevertheless, prior research has not provided a definitive comparison of the relative abundance of these components in terms of dry mass. In our study, despite encountering inter‐donor variability, a heightened composition observed for CHO could be attributed to the considerably larger overall mass of the chorionic membrane in comparison to the amniotic membrane, which may facilitate the extraction of matrix components during the decellularization and digestion processes.

Historically, both AM, CHO or their combination (AM+CHO) have undergone experimental testing, seen clinical use, or been part of clinical trials,^[^ ^17^ ^]^ and the observed differences in growth factor composition between AM and CHO prompted us to establish two distinct extracellular matrix (ECM)‐based extracts. In this current study, we aimed to investigate whether these extracts could yield different results in terms of their physico‐chemical and biological properties. Also, inter‐ and intra‐donor variability,^[^ ^30^, ^31^, ^32^ ^]^ sub regional amniotic tissue differences,^[^ ^33^ ^]^ and the preservation methodology used^[^ ^34^ ^]^ have been reported to impact its therapeutic potential.

In this study, it was crucial to assess inter‐donor variability and analyze the growth factor composition of the tissue. This allowed us to establish a correlation between the production of the biomaterial ink and its biological evaluation. Therefore, we incorporated samples from three distinct placenta donors to ensure a comprehensive evaluation.

**Figure 3:**
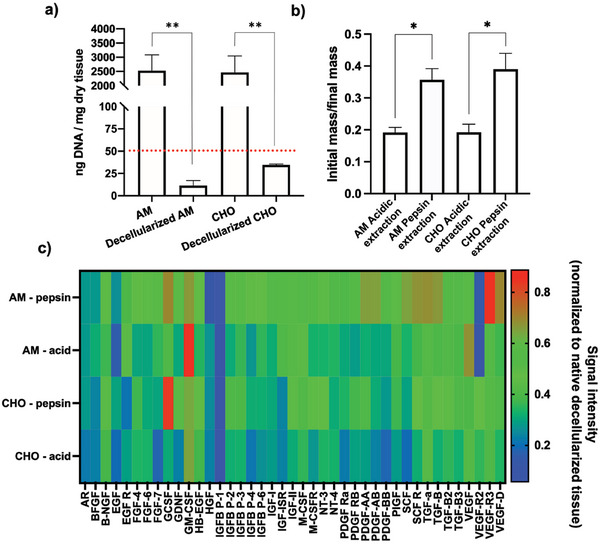
a) Decellularization efficiency evaluation performed by total DNA quantification. Both decellularized AM and CHO showed DNA quantity below 50 ng per mg of dry tissue (*n* = 3 donors; Average ± SD, ** denotes *p* < 0.01). b) Extracellular matrix extraction mass ratio evaluation, considering two extraction methodologies: enzymatic (pepsin) and acidic (3 independent donor tissues, *n* = 3; Average ± SD, * denotes *p* < 0.05). c) Heatmap analysis of a 41 human growth factor array in extracted extracellular matrix obtained using two different methodologies: pepsin enzymatic digestion or acidic extraction (considers 3 independent donor tissues, *n* = 3).

To validate our decellularization methodology, we initially conducted a total DNA quantification for both the AM and CHO. We followed established standards and protocols for this assessment.^[^ ^35^ ^]^ As observed in **Figure** **3**a both AM and CHO showed total DNA concentrations below 50 ng of DNA per mg of total dry mass, considered the lower value to enable an immune reaction following transplantation,^[^ ^36^ ^]^ validating the used methodology for efficient decellularization.

As a subsequent step, our goal was to determine the most effective method for extracting extracellular matrix components. To achieve this, we tested two approaches: acid extraction or enzymatic digestion. As seen in Figure 3b, and for both AM and CHO, the enzymatic digestion showed significant higher mass extraction efficiency than the acidic extraction method, attaining between 35–40% of final mass ratio. Another important aspect we have considered regarded the impact of the extraction method on the retention of existent growth factors. As observed in Figure 3c (and with statistical analysis for each factor on Figure S2A,B, Supporting Information) no major growth factor signal intensity variation was observed, indicating that in both methodologies no relevant differences in terms of growth factor retention could be observed. For 35 factors no significant differences were observed, 5 growth factors were shown to be significantly more expressed for the pepsin digestion and solely for one factor the acidic extraction was significantly higher (Figure S2A,B, Supporting information).

To enable photopolymerization, we followed a previously reported method,^[^ ^27^ ^]^ in which we methacrylated the extracted AM and CHO samples. We then compared these methacrylated extracts with methacrylated type I collagen (CollMA), which served as gold standard for microextrusion bioprinting of endothelial cells.

First preliminary studies using AM‐MA or CHO‐MA alone showed insufficient structural stability for long term cell culture (data not shown). As such, here we have focused on their combination with methacrylated hyaluronic acid (Hya‐MA). Hyaluronic acid is a key matrix component, ubiquitous in the human body, with over 40 years of use in clinics, and it has been extensively demonstrated that the fragments of Hya, obtained by cellular enzymatic degradation and called hyaluronan oligosaccharides, can support vasculogenesis in vivo.^[^ ^37^ ^]^ Furthermore, by utilizing a high molecular weight material, we achieved an increase in viscosity and structural stability. This improvement in material properties enhanced its printability and its ability to support cell maturation over extended periods.

### 2.2 Bioink Formulation and Characterization

**Figure 4:**
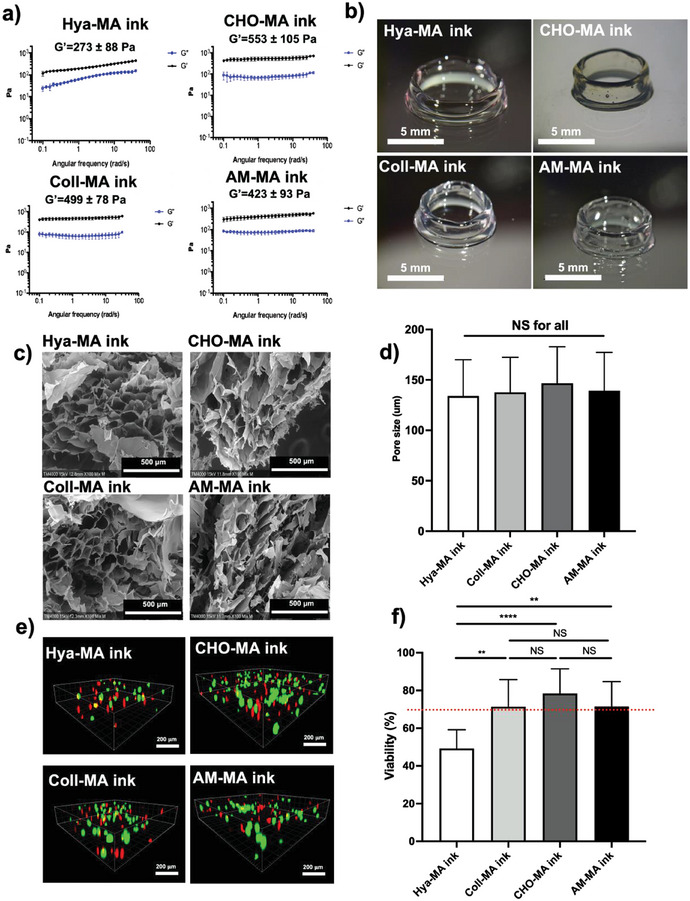
a) Rheological characterization of polymerized methacrylated hyaluronic acid (Hya‐MA ink) at 1.5% (w/v), or Hya‐MA (1.5% w/v) + methacrylated type 1 collagen (Coll‐MA ink) (0.3% w/v) composite, or Hya‐MA (1.5% w/v) + methacrylated chorionic membrane (CHO‐MA ink) (0.3% w/v) composite, or Hya‐MA (1.5% w/v) + methacrylated amniotic membrane (AM‐MA) (0.3% w/v) composite (AM‐MA ink). All compositions contained 0.1% (w/v) of LAP (Lithium phenyl‐2,4,6‐trimethylbenzoylphosphinate) as photoinitiator. Storage modulus (G′) of each formulation is indicated in the figures. (Aver ± SD, *n* = 3). b) Representative brightfield photographs of a printed constructs, 5 mm diameter and 5 mm high hollow tubes, using the 4 different bioinks. All constructs were shown to be stable and regular. c) Scanning electron microscopy (SEM) imaging of the different bioink microporosities and correspondent pore size quantification d) (Aver ± SD, *n* = 20, NS denotes non‐significant). e) Confocal microscopic imaging of the live/dead assay of the four distinct bioinks, composed by Hya‐MA ink, or Coll‐MA ink, or CHO‐MA ink, or AM‐MA ink, at day 1 after bioprinting, and with 5 million mL^−1^ of HUVECs. f) Live/dead assay quantification, expressed as percent viability of HUVECs cultured inside bioprinted Hya‐MA, Coll‐MA, Cho‐MA or AM‐MA bioinks (Aver ± SD, *n* = 8, N = 3, *n* = 4, * and ** denotes *p* < 0.05 or 0.01, respectively, NS denotes non‐significant).

Following the formulation of the biomaterial ink, composed by Hya‐MA alone or combined with CollMA, CHO‐MA or AM‐MA, we then preformed the rheological characterization following composite photopolymerization. As observed in **Figure** **4**a, Hya‐MA gel alone showed a G′ prime of 273 ± 88 Pa, whereas the composite biomaterials inks showed an increase in the storage modulus (G′), ranging from 423 to 553 Pa. We could not observe any significant differences for the composite biomaterials inks. Furthermore, we conducted rheological characterization of both CHO‐MA or AM‐MA alone, as depicted in Figure S4, Supporting Information. In this analysis, the G’ values were observed to fall within the range of 38 to 42 Pa, indicating a relatively low mechanical resilience when these materials are used individually.

Another important parameter to consider in this work regarded the capacity of the developed biomaterial inks to be bioprinted. Here we have focused on microextrusion, due to its current status as the most widely used method for bioprinting, and as offers the major advantages of being able to print very high cell densities and to attain large volumes of production in a shorter amount of time.^[^ ^38^ ^]^ Nonetheless, one disadvantage of microextrusion bioprinting is related to the loss of cellular viability that results from shear stress applied to the extruded bioink.^[^ ^39^ ^]^ As a result, we achieved the successful fabrication of 3D structures, exemplified by the bioprinting of a 5 mm diameter tube that could autonomously support its own weight (as depicted in Figure 4b), all while maintaining cell viability, as demonstrated below. Additionally, and to visually depict the bioprinting process using microextrusion, we have included live videos showcasing CHO‐MA and AM‐MA (see Supporting information videos AM MA V1 and CHO MA V2 for depiction), and respective strut dimension evaluation (see Figure S5, Supporting Information).

Additionally, a scaffold suitable for tissue engineering should have a highly porous structure with interconnected pore network for optimal cell growth and to enable nutrient and metabolic waste flow. When we analyzed the various biomaterial ink formulations using scanning electron microscopy (SEM), which included Hya‐MA alone or composites of Hya‐MA with Coll‐MA, CHO‐MA, or AM‐MA, we were able to observe that all these materials exhibited noticeable microporosity (refer to Figure 4c). Image analysis of the SEM imaging could also show that the average pore size for all formulations was in the range of 100 to 200 µm, and that no significant differences could be establish between the different bioinks (see Figure 4d). The optimal pore size for tissue engineering and tissue regeneration purposes does not have a clear consensus. However, a porosity of between 150 and 350 µm for bone tissue engineering^[^ ^40^ ^]^ or 40–150 µm for skin wound repair^[^ ^41^ ^]^ is generally considered ideal, both in the range of the porosity presented here.

To assess the ability of the developed bioinks to support both the bioprinting process and the survival of HUVECs, we conducted a viability evaluation 24 h after the printing process. As observed in Figure 4e,f, we could show that with the present formulations, and polymerization conditions, HUVECs cells showed an average percent viability of 50, 79, 80 and 78% for Hya‐MA alone or composites of Hya‐MA with Coll‐MA, CHO‐MA or AM‐MA, respectively, at 24 h of culture. For all the composite bioinks the percent viability was higher than 70%, asserting for their biocompatibility in accordance to ISO 10993‐1:2018.

Additionally, and in view of determining the applicability of the developed ECM‐based bioinks to other bioprinting technologies we could demonstrate their efficient use with microvalve (Figure S6, Supporting Information) and laser‐assisted bioprinting (Figure S7, Supporting Information). Based on the results presented, we were able to achieve consistent and replicable bioprinted structures while ensuring the viability of HUVECs.

### 2.3 Amnion and Chorion‐Derived Bioinks Sustain the Bioprinting of Human Endothelial Cells and Support Vasculogenesis

Bioprinting has been the subject of numerous studies in an effort to replicate the vascular structure. The importance of accurately reproducing vasculature in tissue engineering and regenerative medicine is well recognized, as it plays a vital role in the proper functioning and survival of tissues and organs by delivering nutrients and oxygen and removing waste products. Following this premise, we explored the developed bioinks for the bioprinting of human endothelial cells and evaluated their potential to support endothelial cell maturation. Also, as gold standard ink we have focused on type I collagen and hyaluronic acid, to whom human cells have an inherent enzymatic degradation secretome, and where we have previously demonstrated the capacity to sustain vasculogenesis.^[^ ^42^ ^]^ Moreover, given the demonstrated supportive role of fibroblasts in vasculogenesis through their activities in synthesizing and modifying the extracellular matrix (ECM) and aiding in the maturation of endothelial cells,^[^ ^43^ ^]^ they were included in co‐culture with HUVECs within the bioprinted explants.

**Figure 5:**
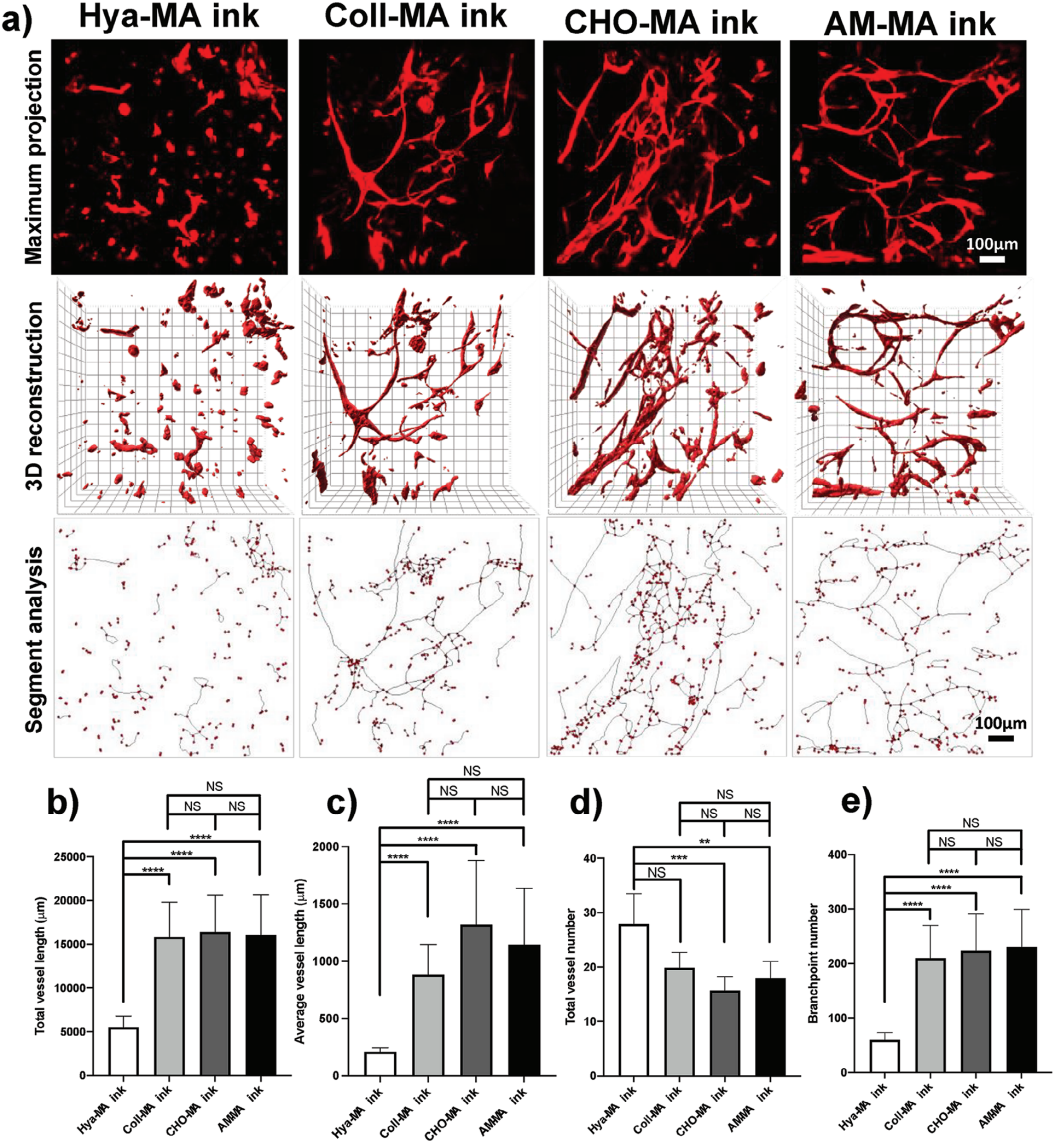
a) Confocal imaging and capillary‐like structure evaluation for RFP^+^ human umbilical vein endothelial cells (HUVECs, red), in 1:1 coculture with human skin fibroblasts, upon bioprinting with 4 distinct biomaterial inks, composed by methacrylated hyaluronic acid (Hya‐MA ink, 1.5% w/v), or Hya‐MA (1.5% w/v) + methacrylated type 1 collagen (0.3% w/v) composite (Coll‐MA ink), or Hya‐MA (1.5% w/v) + methacrylated chorionic membrane (0.3% w/v) composite (CHO‐MA ink), or Hya‐MA (1.5% w/v) + methacrylated amniotic membrane (0.3% w/v) composite (AM‐MA ink), at day 14 post bioprinting. The capillary‐like structure maturation was evaluated by image analysis for the following parameters, b) total vessel length, c) average vessel length, d) total vessel number and e) branchpoint number (Aver ± SD, *n* = 6, N = 3, ** and *** denotes *p* < 0.01 and *p* < 0.001, respectively).

Following a simple grid bioprinting and at 14 days of culture we evaluated the organization and sprouting of RFP^+^ HUVECs, in coculture with human skin fibroblasts. As seen in **Figure** **5**a, and in contrast with Hya‐MA ink, for the 3 ECM‐based composite bioinks one can observe an increase in cell interconnectivity and complexity of the vasculogenic network. Image analysis for total vessel length, average vessel length and branchpoint number have shown a significant increase for the 3 composite bioinks, in relation to Hya‐MA alone (Figure 5b,c,e, respectively). This significant improvement was equally associated with a significant reduction on total vessel number for CHO‐MA and AM‐MA inks, in relation to Hya‐MA alone, demonstrating the significant augmentation of interconnected structures (Figure 5d).

**Figure 6:**
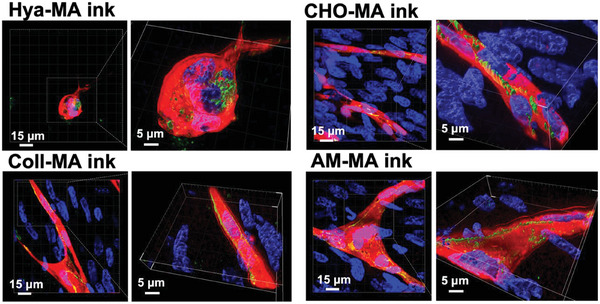
Fluorescence confocal imaging and 3D segmentation of CD31 immunocytochemistry of HUVECs and HSFs bioprinted within Hya‐MA, Coll‐MA, Cho‐MA or AM‐MA bioinks, and cultured for 14 days. HUVECs expressed RFP^+^ (red), CD31 immunostaining (green) and cell nuclei labeled by DAPI (blue).

CD31, also known as platelet endothelial cell adhesion molecule‐1 (PECAM‐1), is abundantly expressed between adjacent endothelial cells in 3D and is a key factor in the maintenance of endothelial cell junctional integrity.^[^ ^44^ ^]^ To assess the 3D cellular organization and maturation of the capillary‐like endothelial structures we proceeded with 3D confocal imaging and reconstruction for the CD31 immunostaining. As observed in **Figure** **6**, in the case of the Hya‐MA biomaterial ink cells organized in large spheroids with the creation of short endothelial cell sprouts. CD31 staining was shown to be disorganized. In contrast, for the three composite biomaterial inks, and in line with a capillary‐like structure organization, one could observe a CD31 staining (green) alongside the tubular structures and accumulated at the intercellular junctions.

A central challenge in the continually advancing field of tissue engineering is the provision of microvasculature to biofabricated tissues where simple diffusive nutrient transport alone becomes insufficient. Consequently, the development of a functional and perfusable microvascular network is imperative, not only for addressing ischemic diseases but also for the ultimate goal of engineering transplantable tissues/organs.^[^ ^45^ ^]^ To achieve this objective, we focused on the validation of these versatile human‐derived biomaterial inks in supporting vasculogenesis.

Despite some differences in composition of ECM components, and growth factor abundance, we did not observe significant disparities in their in vitro capacity to support the maturation of endothelial cells. In a prior study by McQuilling et al., it was demonstrated that unprocessed CHO contained higher levels of certain signalling molecules per cm^2^ when compared to AM. Specifically, the chorion exhibited significantly elevated levels of adiponectin, APN, ANG‐2, bFGF, EG‐VEGF, HGF, IGF‐1, PDGF‐AA, PDGF‐BB, TIMP‐2, and TIMP‐4.^[^ ^26^ ^]^ However, when evaluating the potency of AM and CHO membranes in terms of growth factors per milligram of extracted protein, the researchers found that these membranes exhibited a similar overall composition, with a few exceptions. This study corroborates our findings, suggesting that following processing, both AM and CHO can yield comparable biological effects, at least concerning in vitro vasculogenesis. Nevertheless, the impact on other cellular mechanisms remains incompletely understood and warrants further investigation.

The approach to attain the creation of biomaterial inks following tissue‐specific ECM extraction and applying it to bioprinting of specific tissues has gained huge interest in the recent years. Nonetheless, it has mainly been focused on animal derived tissues, namely porcine,^[^ ^46^, ^47^, ^48^, ^49^, ^50^, ^51^ ^]^ canine^[^ ^52^ ^]^ and murine.^[^ ^52^, ^53^ ^]^ Also, their inherent long gelation time limited the precision and complexity of the shapes created and led to the use of support biomaterials, namely alginate,^[^ ^49^, ^51^, ^53^ ^]^ and gelatin.^[^ ^50^ ^]^ Nevertheless, despite their widespread presence in the scientific field, these biomaterials may encounter challenges in gaining clinical acceptance and widespread use. In contrast, we have focused on combining placental human tissue extracts with a clinically validated polymer, hyaluronic acid,^[^ ^48^ ^]^ improving the capacity of such approach to attain clinical and regulatory acceptance.

The clinical application of placental tissues, AM or CHO, as fresh, freeze‐dried or cryopreserved sheets is presented with several limitations, as difficulty of manipulation due to its inherent fragility and limitations regarding its optimal fixation on injury site for prolonged periods. As such, a novel approach considers the creation of placental tissue‐based hydrogels. Amniotic membrane‐based hydrogels have been previously explored as vehicles for cell delivery. Ryzhuk et al. have developed an AM hydrogel via simple pepsin digestion and have shown comparable results to collagen gels in terms of stem cell proliferation, biocompatibility and also shown no inflammatory or immune reaction following in vivo implantation.^[^ ^54^ ^]^ Nonetheless, these physical hydrogels shown limited mechanical properties in view of their application for biofabrication. In this sense, in a recent report Deus et al. have explored the methacrylation of AM as means to improve its 3D stability. Also, they could demonstrate its applicability for simple printing and then for the culture of human bone marrow stromal cells.^[^ ^21^ ^]^ Nonetheless, they have solely tested AM, and neglected CHO, and have not addressed the bioprinting of such gels.

In contrast to previous studies, our research demonstrates, for the first time, the capability of both amniotic membrane (AM) and chorionic membrane (Cho)‐based bioinks to support the bioprinting, viability, proliferation, and maturation of human endothelial cells, thereby promoting optimal vasculogenesis. By establishing bioinks derived from human sources, we have unlocked a promising avenue for human tissue engineering applications. This significant advancement lays the groundwork for the development of the next generation of bioinks sourced from human materials, bringing us closer to the realization of 3D bioprinting for human tissue transplantation.

Moreover, our findings have broader implications, as we are actively exploring the application of these novel biomaterials for other cell types and tissue engineering endeavors. This exploration of new cell types and applications will open up new avenues for the utilization and further advancement of these bioinks.

## 3 Conclusion and Future Outlook

This work demonstrated for the first time the development of novel biomaterial inks derived from human placental membranes and showcased their application in bioprinting. We successfully isolated and decellularized the amniotic membrane (AM) and chorion (CHO) membranes, and extensively compared their physical, chemical, and biological properties. The ECM‐derived bioinks we developed exhibited remarkable bioprinting capabilities and supported the viability of human cells, demonstrating their potential to sustain vasculogenesis, a hallmark for tissue engineering and regenerative medicine applications. Moving forward, future studies will focus on validating these biomaterials in in vivo models to evaluate their capacity for tissue repair and integration. Overall, this research represents a significant advancement in the field of biomaterial development, offering a new avenue for the creation of functional tissues using human placental membranes as a source.

## 4 Experimental Section

### Placental Membrane Dissociation

Human placental samples were obtained from consenting mothers, following caesarean section, under an established protocol with the Bordeaux Hospital (France) and approved by the institutional review board. All donors had mono‐fetal pregnancies and were seronegative for human immunodeficiency virus (HIV) 1 or 2 and for hepatitis B and C viruses. Tissues were kept in ice‐cold phosphate buffered saline (PBS 1X, Gibco) supplemented with 100 U mL^−1^ penicillin and 100 µg mL^−1^ streptomycin (Gibco). The next day, negative HIV serologies 1 and 2 were confirmed using an immunochromatography test (ALERE, ref: 7D2346) prior to dissection.

First, the umbilical cord was removed and the tissues rinsed with sterile PBS at least six times to remove as much blood as possible. The placenta and its membranes were then placed on a large cutting board and the membranes were cut using a surgical blade and the amnion and chorion of each sheet were then manually separated and stored at −20 °C until needed.

### Growth Factors Quantification

A human growth factor antibody array (ab134002, Abcam, UK) was used to assess 41 human growth factor profile of the candidate biomaterial inks. This work investigated the eventual difference in human factor content between AM and CHO, and between the ECM extraction processes (pepsin or acid). The targets considered were Amphiregulin, bFGF, EGF, EGF R, FGF‐4, FGF‐6, FGF‐7, GCSF, GDNF, GM‐CSF, HB‐EGF, HGF, IGFBP‐1, IGFBP‐2, IGFBP‐3, IGFBP‐4, IGFBP‐6, IGF‐I, IGF‐I SR, IGF‐II, M‐CSF, M‐CSF R, beta‐NGF, NT‐3, NT‐4, PDGF Ra, PDGF Rß, PDGF‐AA, PDGF‐AB, PDGF‐BB, PLGF, SCF, SCF R, TGF‐alpha, TGF‐beta, TGF‐beta2, TGF‐beta3, VEGF‐A, VEGF R2, VEGF R3 and VEGF‐D.

In short, freeze‐dried AM or CHO, from 3 distinct donors, were weighted and immersed in sterile PBS at 2 mg mL^−1^. Samples were mixed using a Ultra Turrax blender (IKA, France), using 3 cycles and at 4 °C. The obtained extract was then dissolved to 0.2 mg mL^−1^ using the suppliers blocking buffer and the suppliers protocol was applied. Chemiluminescence signals were acquired using the ImageQuant LAS 4000 mini (GE, USA), quantified with the ImageQuant TL software (v 7.0, GE, USA) and expressed as normalized signal intensity.

### Label‐Free Quantitative Proteomics

Three independent human sourced biomaterials from decellularized amnion and chorion were analyzed by mass spectrometry to decipher the protein composition. 10 µg of proteins were loaded on a 10% acrylamide SDS‐PAGE gel and proteins were visualized by Colloidal Blue staining. Migration was stopped when samples had just entered the resolving gel and the unresolved region of the gel was cut into only one segment. The steps of sample preparation and protein digestion by the trypsin were performed as previously described.^[^ ^55^ ^]^ NanoLC‐MS/MS analysis were performed using an Ultimate 3000 RSLC Nano‐UPHLC system (Thermo Scientific, USA) coupled to a nanospray Orbitrap Fusion Lumos Tribrid Mass Spectrometer (Thermo Fisher Scientific, California, USA). Each peptide extracts were loaded on a 300 µm ID × 5 mm PepMap C_18_ precolumn (Thermo Scientific, USA) at a flow rate of 10 µL min^−1^. After a 3 min desalting step, peptides were separated on a 50 cm EasySpray column (75 µm ID, 2 µm C18 beads, 100 Å pore size, ES903, Thermo Fisher Scientific) with a 4–40% linear gradient of solvent B (0.1% formic acid in 80% ACN) in 57 min. The separation flow rate was set at 300 nL min^−1^. The mass spectrometer operated in positive ion mode at a 1.9 kV needle voltage. Data were acquired using Xcalibur 4.4 software in a data‐dependent mode. MS scans (m/z 375–1500) were recorded at a resolution of R = 120 000 (@ m/z 200), a standard AGC target and an injection time in automatic mode, followed by a top speed duty cycle of up to 3 s for MS/MS acquisition. Precursor ions (2 to 7 charge states) were isolated in the quadrupole with a mass window of 1.6 Th and fragmented with HCD@28% normalized collision energy. MS/MS data were acquired in the Orbitrap cell with a resolution of R = 30 000 (@m/z 200), a standard AGC target and a maximum injection time in automatic mode. Selected precursors were excluded for 60 s. Protein identification and Label‐Free Quantification (LFQ) were done in Proteome Discoverer 2.5. MS Amanda 2.0, Sequest HT and Mascot 2.5 algorithms were used for protein identification in batch mode by searching against a Uniprot *Homo sapiens* database (75 796 entries, release September 3, 2020). Two missed enzyme cleavages were allowed for the trypsin. Mass tolerances in MS and MS/MS were set to 10 ppm and 0.6 Da. Oxidation (M) and acetylation (K) were searched as dynamic modifications and carbamidomethylation (C) as static modification. Peptide validation was performed using Percolator algorithm^[^ ^56^ ^]^ and only “high confidence” peptides were retained corresponding to a 1% false discovery rate at peptide level. Minora feature detector node (LFQ) was used along with the feature mapper and precursor ions quantifier. The normalization parameters were selected as follows : 1) Unique peptides 2) Precursor abundance based on intensity 3) Normalization mode : total peptide amount 4) Protein abundance calculation : summed abundances 5) Protein ratio calculation : pairwise ratio based and 6) Hypothesis test : *t*‐test (background based). Quantitative data were considered for master proteins, quantified by a minimum of 2 unique peptides, a fold changes above 2 and found similarly regulated in the 3 independent biological replicates and a statistical p‐value adjusted using Benjamini‐Hochberg correction for the FDR lower than 0.05. Data was expressed as abundance of the identified ECM proteins through the rank and over the total signal of identified proteins. Quantitative date was expressed as median with interquartile range. Presented data is representative of 3 independent amnion and 3 independent chorion membrane extractions.

### Decellularization

AM and CHO were first decellularized by being soaked in Tris/EDTA for 2 min at 37 °C, then rinsed thrice in phosphate buffer saline (PBS) for 5 min. They were then steeped in a decellularization solution composed of 4.9 mg mL^−1^ CHAPS (CAS: 75621‐03‐3, Sigma Aldrich, France), 4.8 mg mL^−1^ sodium hydroxide (NaOH), 7.3 mg mL^−1^ Ethylenediaminetetraacetic acid tetrasodium salt (EDTA) and 58.4 mg mL^−1^ sodium chloride (NaCl), for 7 h at room temperature, under gentle agitation. The membranes were then rinsed with PBS for 5 min, then with distilled water overnight, at room temperature, under gentle agitation.

Decellularization efficiency was evaluated using a DNA quantification assay and the acceptance levels were determined following the standards established by the work of Crapo and colleagues,^[^ ^57^ ^]^ with maximum acceptable levels at 50 ng of DNA per mg of dry tissue. The procedure used consisted on weighing 10 mg of freeze dried decellularized or native tissue and then using the Qiamp DNA mini kit (Qiagen, France), and following the suppliers protocol, to attain DNA extraction. DNA quantification was performed using a nanodrop spectrophotometer (Scientec P330, France).

### ECM extraction

Two ECM extraction methods were compared in terms of growth factor preservation.

The first method is the most used when it comes to ECM solubilization. Following previous reports,^[^ ^58^ ^]^ AM or CHO were soaked in a solution containing pepsin (0.1 mg mg^−1^ of dry tissue) and 0.1 m hydrochloric acid (HCl) at constant stir for 48 h at 25 °C. Then, neutralized with NaOH 0.1 m until pH 7.4, for pepsin inactivation, and then dialyzed (12–14 kDa, Sigma‐Aldrich) for 5 days against sterile mQ water. The obtained extract was then freeze‐dried, recovered and stored at −20 °C until further use.

The second method was reported by previous reports,^[^ ^59^, ^60^ ^]^ and considered an acetic acid based extraction, reported to better preserve the structure of peptides and growth factors present in the matrix. Briefly, decellularized AM or CHO membranes were soaked in 0.5 m acetic acid (sample mass/solution volume ratio of 1:50 w/v) for 3 days with a gentle stirring. Then, the mix was centrifuged at 21 200 g for 30 min at 4 °C and the supernatants collected and kept at 4 °C. The remaining precipitate was re‐extracted using 0.5 m acetic acid (sample/solution ratio of 1:30 w/v) during 2 days and with a gentle stirring, followed by a centrifugation at 21 200 g for 30 min at 4 °C. Both extracts were combined and dialyzed against 10 volumes of 0.1 m acetic acid in a dialysis membrane with of 12–14 kDa molecular weight cut‐off, 24 h at 4 °C. Then, the solution was dialyzed against 10 volumes of mQ water for 24 h at 4 °C and samples were freezed at −80 °C, freeze dried, recovered, and stored at −20 °C until further use.

### Biomaterial Ink Formulation

Ink formulation was optimized with the aim to have fine rheological properties, for efficient bioprinting, while maintaining the biological added value of AM and CHO.

As to attain optimal rheological properties and to enable photopolymerization both hyaluronic acid (Hya), type I collagen (Coll) AM and CHO were methacrylated as follows.

Methacrylation of Hya. The procedure was as previously reported.^[^ ^27^ ^]^ Briefly, one gram of Hya from Streptococcus Equi (1.5–1.8 × 10E6 Da, Sigma‐Aldrich, France) was dissolved in 75 mL of mQ water, overnight at room temperature. Then, 50 mL of dimethylformamide (Sigma‐Aldrich, France) was added and pH was adjusted to 8 with NaOH 1 m. 1.12 mL of methacrylic anhydride (Sigma‐Aldrich) was added to the solution, under stirring, the pH adjusted to 8 and left under stirring overnight. Then, 50 mL of NaCl 5 m was added and the volume adjusted to 500 mL using mQ grade water. The solution was then filtered using a 0.2 micron filter and then dialyzed (12–14 kDa, Sigma‐Aldrich) for 5 days against 5 L mQ water and with two daily water changes. The polymer was freeze‐dried, recovered and stored at −20 °C until further use. Hyaluronic acid methacrylate (Hya‐MA) stock solutions were prepared by dissolving the polymer at 3% (w/v) in DMEM HG (Gibco) and then stored at 4 °C until further use. Methacrylation degree was determined using nuclear magnetic resonance (NMR). Briefly, for 1H‐NMR a 1% (w/v) solution of HAMA was prepared in D2O (Sigma‐Aldrich) and analyzed at 25 °C on a Bruker Avance I NMR spectrometer operating at 400 MHz. 1H NMR spectra were recorded with an acquisition time of 4 s, a relaxation delay of 2 s and 64 scans. Methacrylate modification was determined by integration of the vinyl singlets relative to the ring of hyaluronic acid and was observed to be 22 ± 4% (average ± SD, *n* = 4, one key example of NMR analysis can be found in Figure S3, Supporting Information).

Methacrylation of Cho, AM or Coll. The procedure was adapted from previous report.^[^ ^27^ ^]^ Briefly, the pH of type I collagen from bovine skin (Coll, Sigma‐Aldrich), Cho or AM solutions were adjusted to 10 using 2N NaOH and kept on ice, under mild agitation. Then, methacrylic anhydride at a molar ratio of 5:1 (with respect to number of lysine amine groups present in the native extract or collagen) was added subsequently drop‐wise and left to react for 4 h. Then, the obtained solution was dialyzed (12–14 kDa, Sigma‐Aldrich) for 5 days against 5 L mQ water and with 2 daily water changes. The obtained solution was then freeze‐dried for 5 days, and the polymer recovered and stored at −20 °C until further use. The methacrylated stock solutions were prepared at 6 mg mL^−1^ in 0.02 m Acetic acid and stored at 4 °C until further use.

Methacrylation degree was determined using the Tri‐nitro benzene sulfonic acid (TNBS) assay. Briefly, neutralized native solutions were dissolved in 1 mL of carbonate buffer (0.1 m, pH 8.5) at 0.1 mg mL^−1^ NaHCO3 (pH 8.5). Then to 500 µL of native or methacrylated solutions, 250 µL of a 1:500 dilution, was added in carbonate buffer, of TNBSA solution (5% w/v, Sigma‐Aldrich) and let to react for 2 h at 37 °C. The reaction was then stopped by the addition of 250 µL of 10% (w/v) of SDS (Sigma‐Aldrich) and 125 µL of 1 m HCl. The OD_335_ of both the blanc, native and methacrylated solutions was determined using a UV–vis spectrophotometer (Nanodrop, ThermoFisher). The percentage of remaining free primary amines of methacrylated polymer was calculated in relation to the total amount of the pristine polymer. The degree of methacrylation was then determined using the loss of primary amines percentage loss. The degree of methacrylation was 53 ± 17% for Coll‐MA, 36 ± 14% for AM‐MA and 31 ± 6% for Cho‐MA (Aver ± SD, *n* = 3).

From this based biomaterials, 4 different biomaterial inks were prepared. The positive control, 0.3% (w/v) Coll‐MA with 1.5% (w/v) Hya‐MA, was established following the previous reports.^[^ ^27^ ^]^ As means to validate the new bioinks and to enable to compare them with Coll‐MA Hya‐MA was set again at 1.5% (w/v) and both AM‐MA and CHO‐MA at 0.3% (w/v). The negative control considered Hya‐MA at 1.5% (w/v) alone.

Briefly, Hya‐MA/Coll‐MA, Hya‐MA/AM‐MA, Hya‐M/CHO‐MA, 1.5 and 0.3% (w/v) respectively, and Hya‐MA 1.5%, composite inks were prepared by diluting and neutralizing the 0.6% (w/v) stock solution of Coll‐MA, AM‐MA or CHO‐MA with 0.1 M NaOH. For the composite formulations, Hya‐MA 4% (w/v) was then added to the neutralized solutions to attain a final 1.5% (w/v) of Hya‐MA. Lithium phenyl (2,4,6‐trimethylbenzoyl) phosphinate (LAP, LTI Chemicals, Japan) photo initiator was added to a final concentration of 0.1% (w/v) and the final volume adjusted using DMEM medium (Gibco, France). Solutions were kept on ice, and protected from light, before bioprinting. Their rheology was then assessed.

### Rheology

The different hydrogel formulations were prepared, loaded and directly polymerized (2w, 365 nm UV source and an exposure time of 1 min) in a Kinexus pro+ rheometer (Malvern Instruments, UK) and stabilized at 37 °C. The storage and loss moduli (G′ and G″, respectively), were determined by performing frequency sweeps at a constant strain (0.1%) in a 0.1–100 rad s^−1^ angular frequency range. Measurements were performed in triplicate and at 37 °C.

### Bioprinting Procedure

In order to determine the suitability of the developed bioinks for bioprinting, this work focused on using microextrusion technology, the most commonly used bioprinting method. This work optimized bioprinting conditions such as displacement speed, pressure, distance to substrate, tip diameter, geometry, and interlayer thickness to maximize cell survival and maintain accurate geometry.

For printability assessment a design of 5 mm diameter, 4 mm high, cylinder was followed, and using a 3D Discovery bioprinter (RegenHU, Switzerland). ≈200 uL of each biomaterial ink formulation was loaded into a 2 mL syringe bearing a conic microextrusion tip of 0.21 mm diameter (RegenHU, Switzerland). Each layer was polymerized (2w, 365 nm UV source and an exposure time of 10 s).

To evaluate the suitability of the bioinks for microextrusion bioprinting, this work employed a 3D Discovery bioprinter (RegenHU, Switzerland), loaded with ≈200 uL of each bioink formulation into a 2 mL syringe, bearing a conic microextrusion tip of 0.21 mm diameter, and printed a 5 × 5 mm grid design with 2.5 mm spacing in a 6 well plate (Sarstedt, Germany). A 4–5 millipascal air pressure was applied and stacked the design three times. The printed samples were exposed to a 365 nm, 2 W photopolymerization diode for 15 s between layers and a final 60 s to ensure crosslinking and 3D stability. The samples were then cultured in EGM‐2 MV medium (Lonza, France) until specified timepoints.

### Porosity

As to determine the micro porosity of the developed hydrogels this work performed the imaging of the different biomaterial inks following polymerization, hydration, subsequent snap freezing and freeze drying. Briefly, following polymerization samples were incubated using ultrapure water for 1 h, then snap frozen using liquid nitrogen and freeze dried (0.25 mBar, −80 °C, Benchtop Pro, SP Scientific, France). Then samples were fractured and placed in carbon tape and then gold sputtered using a sputter coater (EMscope SC500, UK). Electron scanning microscopy (SEM) was performed using a TM 4000 Plus (Hitachi, Japan) at 15 Kv and microporosity quantified using the ImageJ (Fiji, v2.1.0) software.

### Cell Culture

Human umbilical vein endothelial cells (HUVECs) were isolated as previously described.^[^ ^61^ ^]^ HUVECS were cultured using IMDM medium (Gibco, France), with 20% foetal bovine serum (FBS) and endothelial cell growth supplement (ECGS)/Heparin (PromoCell, France), until passage 9. Also, as to enable their visualization by fluorescent microscopy HUVECs were transduced at cell passage 2 with lentiviral vectors bearing the RFP gene, under the EF1a promoter sequence (Vectalys, France).

Human skin fibroblasts (HSF) were isolated as previously described,^[^ ^62^ ^]^ and cultured in DMEM:F12 (Gibco, France), 20% (v/v) FBS, until passage 9.

### Cell Viability Evaluation

Live‐dead assay was performed to assess the cell viability after being printed in the four formulations. Printed samples were stained with calcein‐AM (“live,” 1 ​µL mL^−1^, Invitrogen) and ethidium homodimer (“dead,” 4 ​µL mL^−1^, Invitrogen) for 20 min. They were then rinsed with PBS and analyzed by confocal microscopy (SPE7, Leica Microsystems, Germany). Image analysis for the determination of the percentage of viable cells was performed using ImageJ by determining the total number of cells per field (cells positive for calcein + cells positive for ethidium homodimer), obtained by a 2D projection of a homogeneous acquired confocal volume. Percent (%) viability was therefore determined as the percentage of “live” cells (cells positive for calcein, *n* = 8 replicates, N = 3).

### Vasculogenesis Evaluation

As to evaluate the capacity of the developed biomaterial inks to sustain vasculogenesis HUVECs and HSFs (both at 20 million mL^−1^) were bioprinted using the four different bioink formulations, and cultured them for 21 days. Constant and representative volumes of the samples were acquired by confocal microscopy, and analyzed as previously described by Krishnan et al..^[^ ^63^ ^]^ Briefly, the vessels were recreated using Amira software (Thermo Fisher, Germany) by piling the stacks of the acquired volume. The image was filtered, and a skeleton was created for further analysis of vessel lengths and branchpoints (*n* = 6 replicates, N = 3).

### Immunocytochemistry

The same samples used for angiogenesis analysis were stained for CD31, in order to assess the phenotypic maturation of the printed HUVECs, as follows: samples were fixed using 4% (w/v) paraformaldehyde for 20 min at room temperature, and washed with PBS. They were then frozen at −20 °C for 1 h, in order to increase the permeation of the gel. After being thawed at 37 °C for 15 min, the PBS was removed from the sample, which was then incubated with Triton X‐100 (0.1% v/v) for 15 min. After being washed with PBS, samples were blocked with 2.5% (w/v) bovine serum albumin for 1 h, then incubated with rabbit Anti‐CD31 polyclonal (ab28364, Abcam, UK), at 1:200 in 1% BSA (w/v), overnight at 4 ​°C. Samples were then washed with PBS, and incubated with Alexa 488 goat anti‐rabbit (A11008, Invitrogen), at 1:400 in 0.5% BSA (w/v), during 4 ​h at room temperature. After being washed with PBS, samples were incubated with DAPI (4′, 6′‐diamidino‐2‐phenylindole) (Vector Labs) at 1 ​µg mL^−1^ for 10 ​min ​at room temperature. After 2 additional washes with PBS, samples were mounted using Vectashield (Vector) and analyzed by confocal microscopy.

## Conflict of Interest

The authors declare no conflict of interest.

## Supporting information

Supporting Information

Supplemental Video 1

Supplemental Video 2

## Data Availability

The data that support the findings of this study are available from the corresponding author upon reasonable request.
