# Supplementary material for: Harnessing Human Placental Membrane‐Derived Bioinks: Characterization and Applications in Bioprinting and Vasculogenesis
Source: Adv Healthc Mater. 2023 Nov 20;13(6):2303370. doi: 10.1002/adhm.202303370 (PMC11469061; doi:10.1002/adhm.202303370)
Supplement: Supplementary file 1 — Supporting Information [file ADHM-13-2303370-s002.pdf]

ADVANCED HEALTHCARE MATERIALS

Supporting Information

for *Adv. Healthcare Mater.*, DOI 10.1002/adhm.202303370

Harnessing Human Placental Membrane-Derived Bioinks: Characterization and Applications in Bioprinting and Vasculogenesis

*Léo Comperat, Lise Chagot, Sarah Massot, Marie-Laure Stachowicz, Nathalie Dusserre, Chantal Médina, Théo Desigaux, Jean-William Dupuy, Jean-Christophe Fricain and Hugo Oliveira**

Supporting Information

Harnessing human placental membrane-derived bioinks: Characterization and applications in bioprinting and vasculogenesis

*Léo Comperat1,2, Lise Chagot1,2, Sarah Massot1,2, Marie-Laure Stachowicz1,2, Nathalie Dusserre1,2, Chantal Médina1,2, Théo Desigaux1,2, Jean-William Dupuy3, Jean-Christophe Fricain1,2,4, Hugo Oliveira1,2 **

1 University of Bordeaux, Tissue Bioengineering, U1026, F-33076 Bordeaux, France
2 Inserm U1026, Tissue Bioengineering, ART BioPrint, F-33076 Bordeaux, France
3 CHU Bordeaux, Services d'Odontologie et de Santé Buccale, F-33076 Bordeaux, France

E-mail: hugo.de-oliveira@inserm.fr

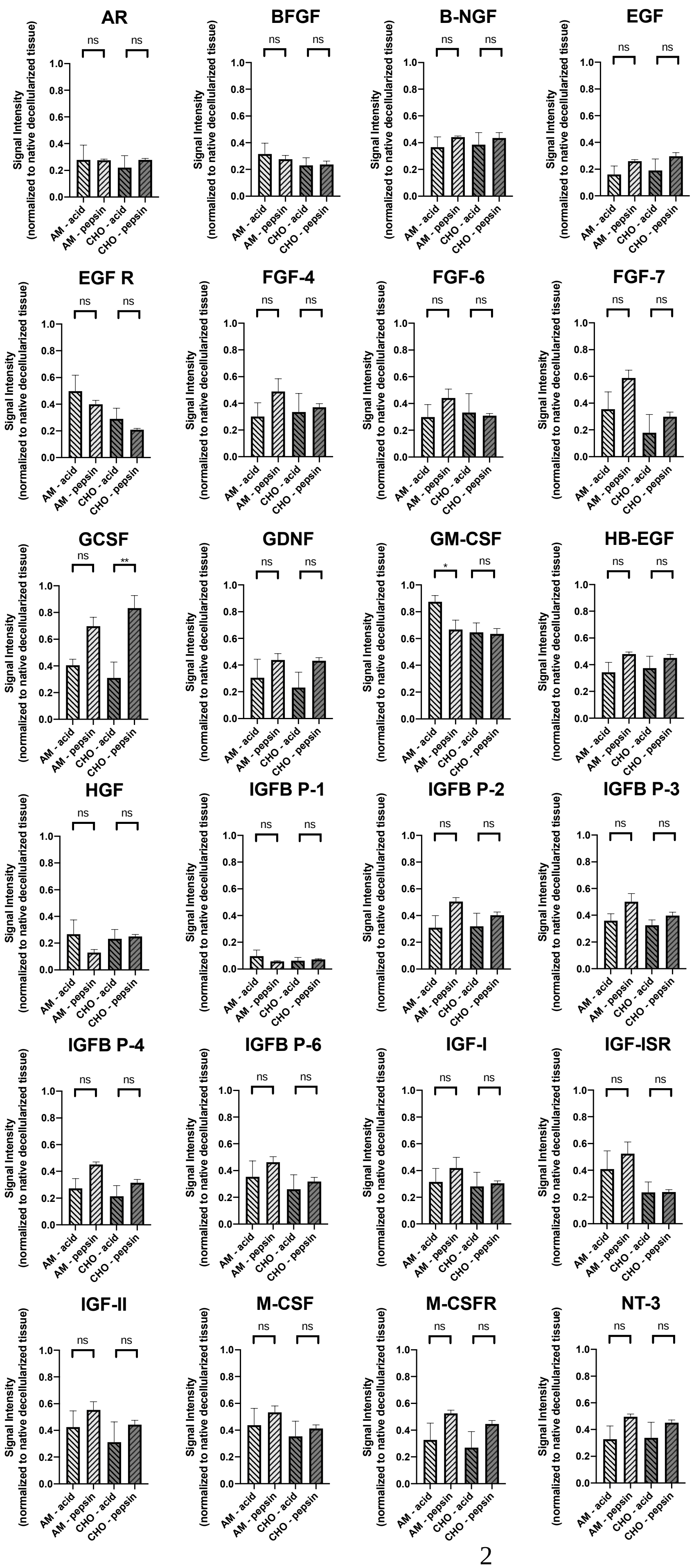
AR
BFGF
B-NGF
EGF
EGF R
FGF-4
FGF-6
FGF-7
GCSF
GDNF
GM-CSF
HB-EGF
HGF
IGFB P-1
IGFB P-2
IGFB P-3
IGFB P-4
IGFB P-6
IGF-I
IGF-ISR
IGF-II
M-CSF
M-CSFR
NT-3
Signal Intensity (normalized to native decellularized tissue)
AM - acid
AM - pepsin
CHO - acid
CHO - pepsin
ns
*
**

Figure S2 A- Relative quantification of 41 human growth factors present in decellularized human amniotic membrane (AM) or decellularized human chorion (CHO) extracts, obtained using an acidic or enzymatic (pepsin) methodology, and expressed as signal intensity normalized to the correspondent native decellularized tissue. Average values compiled from 3 independent membrane donors (n=3). * and ** denote $p<0.05$ and $p<0.01$, respectively. ns denotes non-significant.

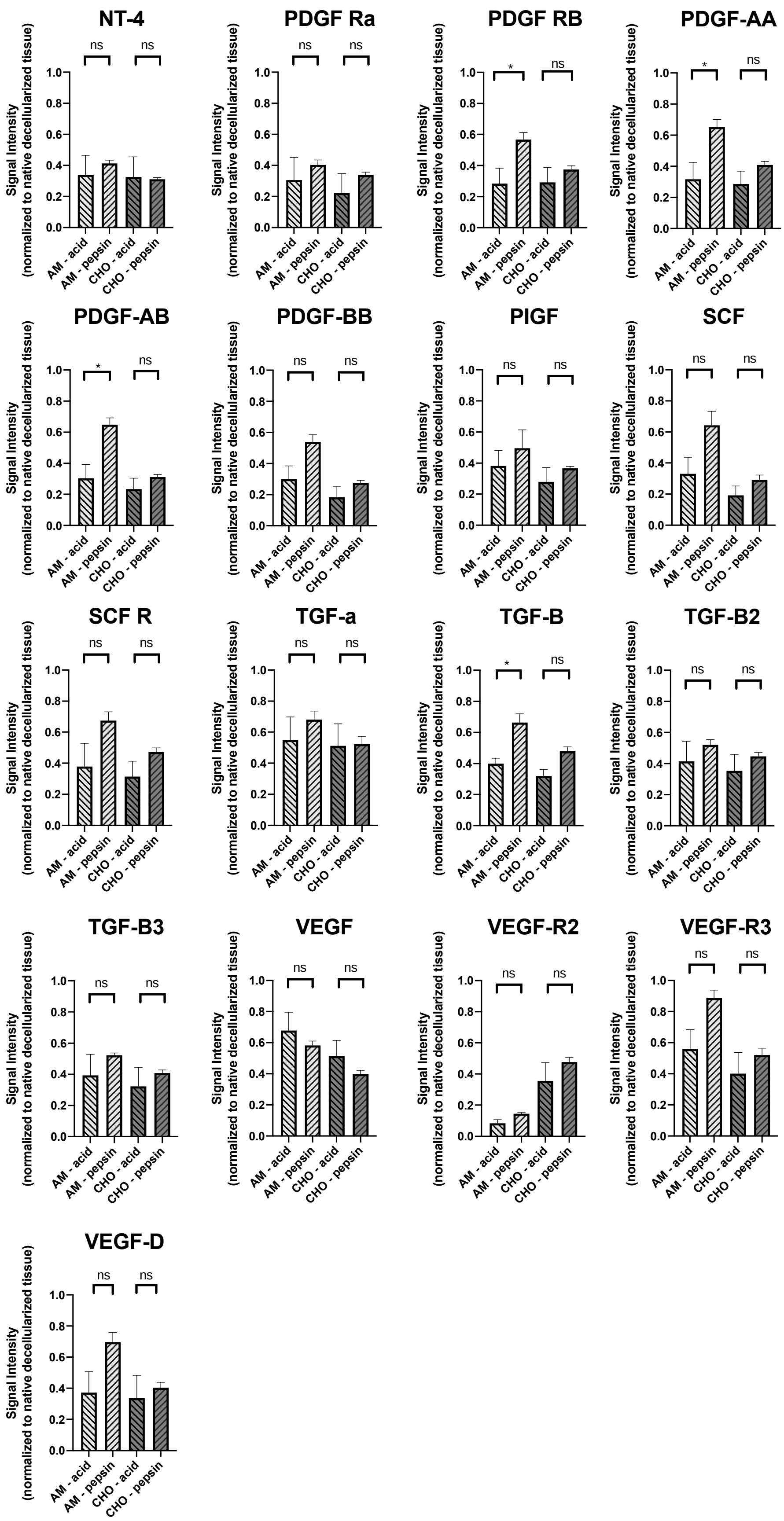

Figure S2 B- Relative quantification of 41 human growth factors present in decellularized human amniotic membrane (AM) or decellularized human chorion (CHO) extracts, obtained using an acidic or enzymatic (pepsin) methodology, and expressed as signal intensity

normalized to the correspondent native decellularized tissue. Average values compiled from 3 independent membrane donors (n=3). * and ** denote p<0.05 and p<0.01, respectively. ns denotes non-significant.

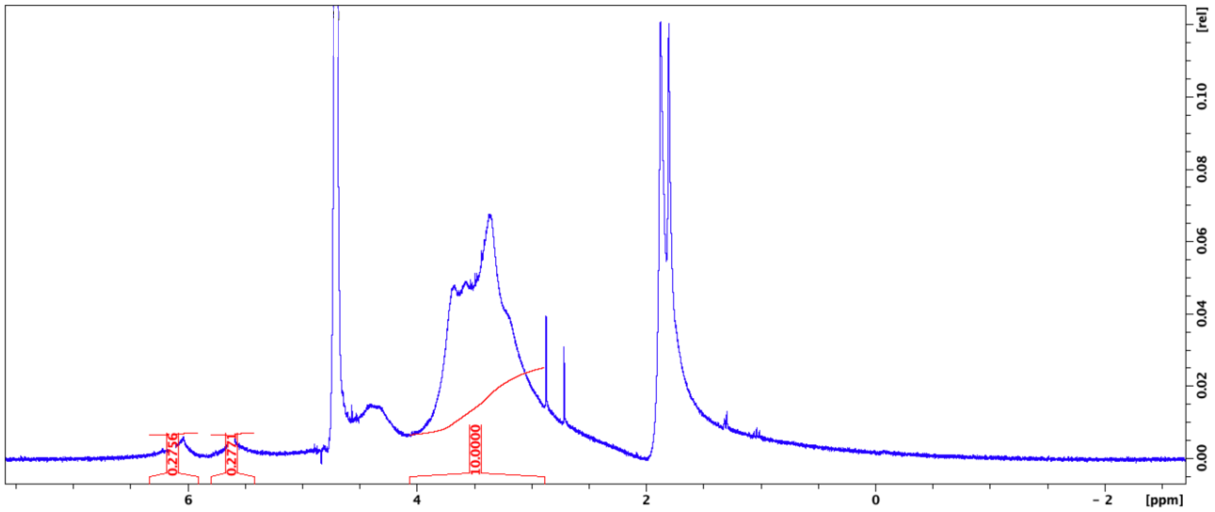

Figure S3- ^{1}H NMR spectrum of methacrylated hyaluronic acid (HAMA) in D_2O. Methacrylate modification as determined by integration of the vinyl singlets (1H each, shaded green) relative to the ring of hyaluronic acid (10H, shaded grey).

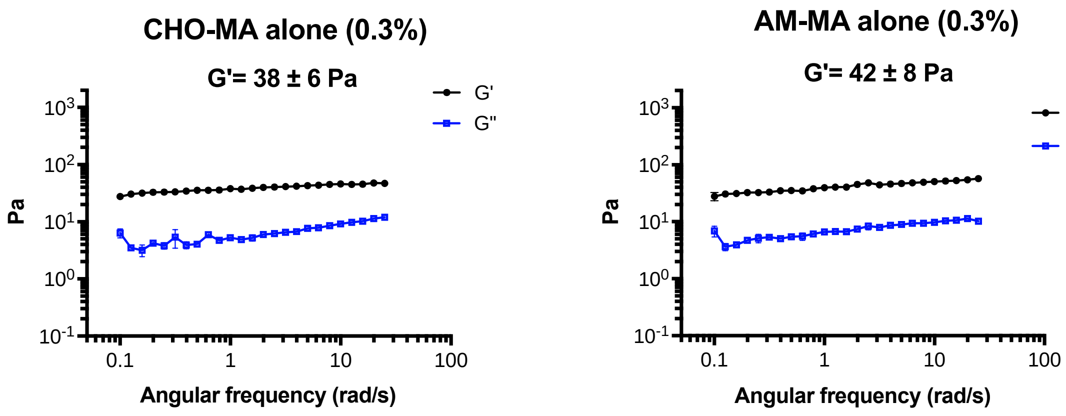

Figure S4- Rheological characterization of polymerized methacrylated chorionic membrane (CHO-MA alone) (0.3% w/v), or methacrylated amniotic membrane (AM-MA alone) (0.3% w/v). All compositions contained 0.1 % (w/v) of LAP (Lithium phenyl-2,4,6-trimethylbenzoylphosphinate) as photoinitiator. Storage modulus (G') of each formulation is indicated in the figures (Aver±SD, n=3).

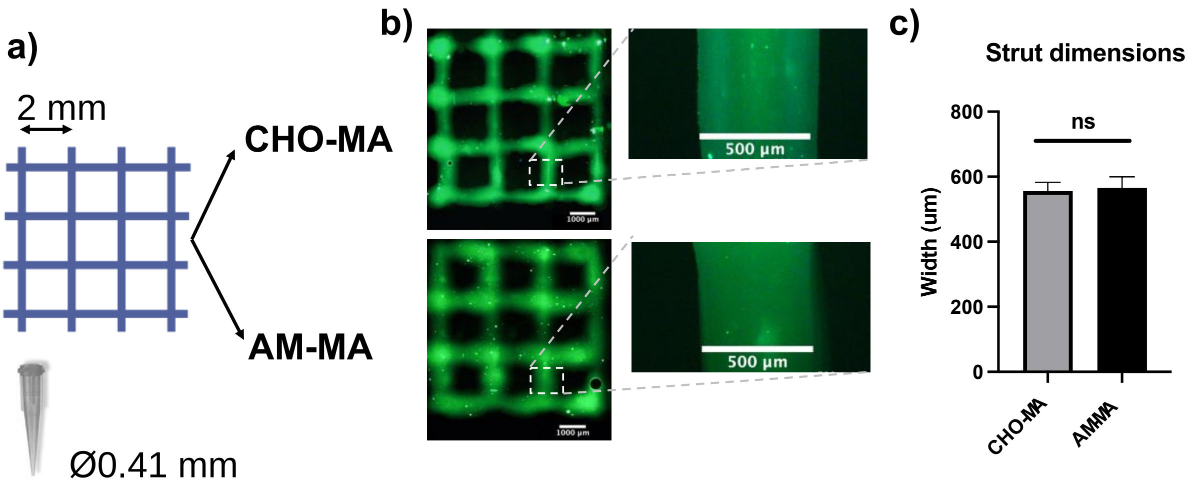

Figure S5- a) Schematic representation of the tested geometry, consisting on a 4 by 4 grid, with 2mm interline distance and using a 0.41 mm diameter conical tip. b) Fluorescence microscopy images of grid geometries fabricated using 2 distinct biomaterial inks, Hya-MA (1.5% w/v) + methacrylated chorionic membrane (0.3% w/v) composite (CHO-MA), or Hya-MA (1.5% w/v) + methacrylated amniotic membrane (0.3% w/v) composite (AM-MA ink). Both inks contained 0.15 % w/v of photoinitiator LAP and 0.1% of FITC Dextran (70 KDa, D1823, ThermoFisher). c) Strut width was determined by image analysis using ImageJ and plotted as Width (Aver±SD, ns denotes non-significant).

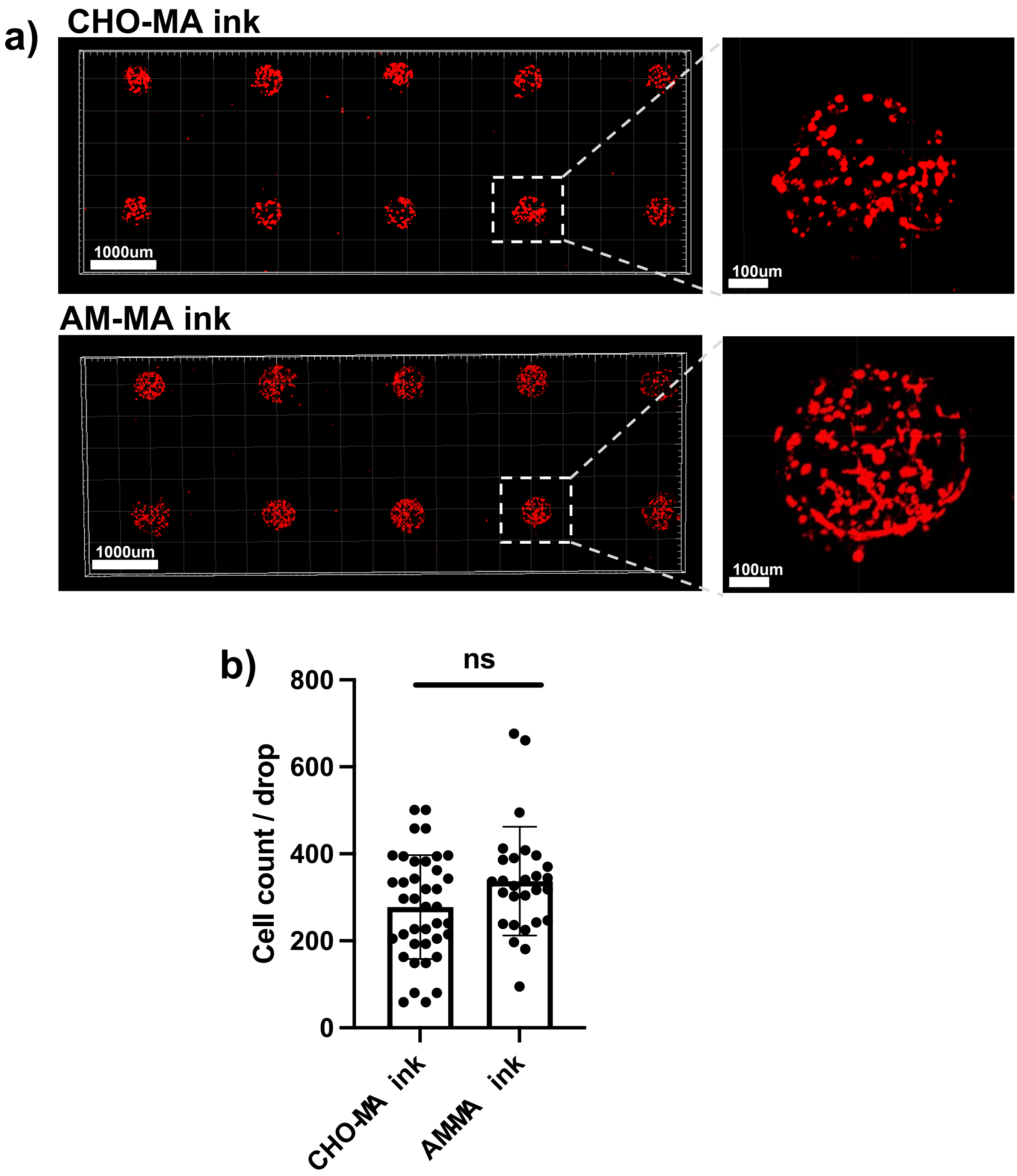

Figure S6- a) Confocal imaging of microvalve bioprinted RFP+ human umbilical vein endothelial cells (HUVECs, red), with 2 distinct biomaterial inks, composed by methacrylated chorionic membrane (0.3% w/v, CHO-MA ink), or methacrylated amniotic membrane (0.3% w/v, AM-MA ink), one day post bioprinting. Both inks contained 0.15 % w/v of photoinitiator LAP. Bioprinting considered the use of the 3D Discovery bioprinting platform (RegenHU, SWZ) with a 300 µm diameter microvalve, HUVECs were printed at 20 millions/mL in 0.3% w/v CHO-MA or AM-MA inks with pressure= 0.020 MPa, valve opening time at 150 µs and a distance to the substrate of 2 mm. Droplet interdistance was of 1.5 mm and followed by a 1 min 365 nm UV photopolymerization. Cells were cultured in EGM-2

MV medium (Lonza, France). b) Cell number by droplet was quantified using image analysis (Imaris) and plotted as cell count per drop (Aver±SD, ns denotes non-significant).

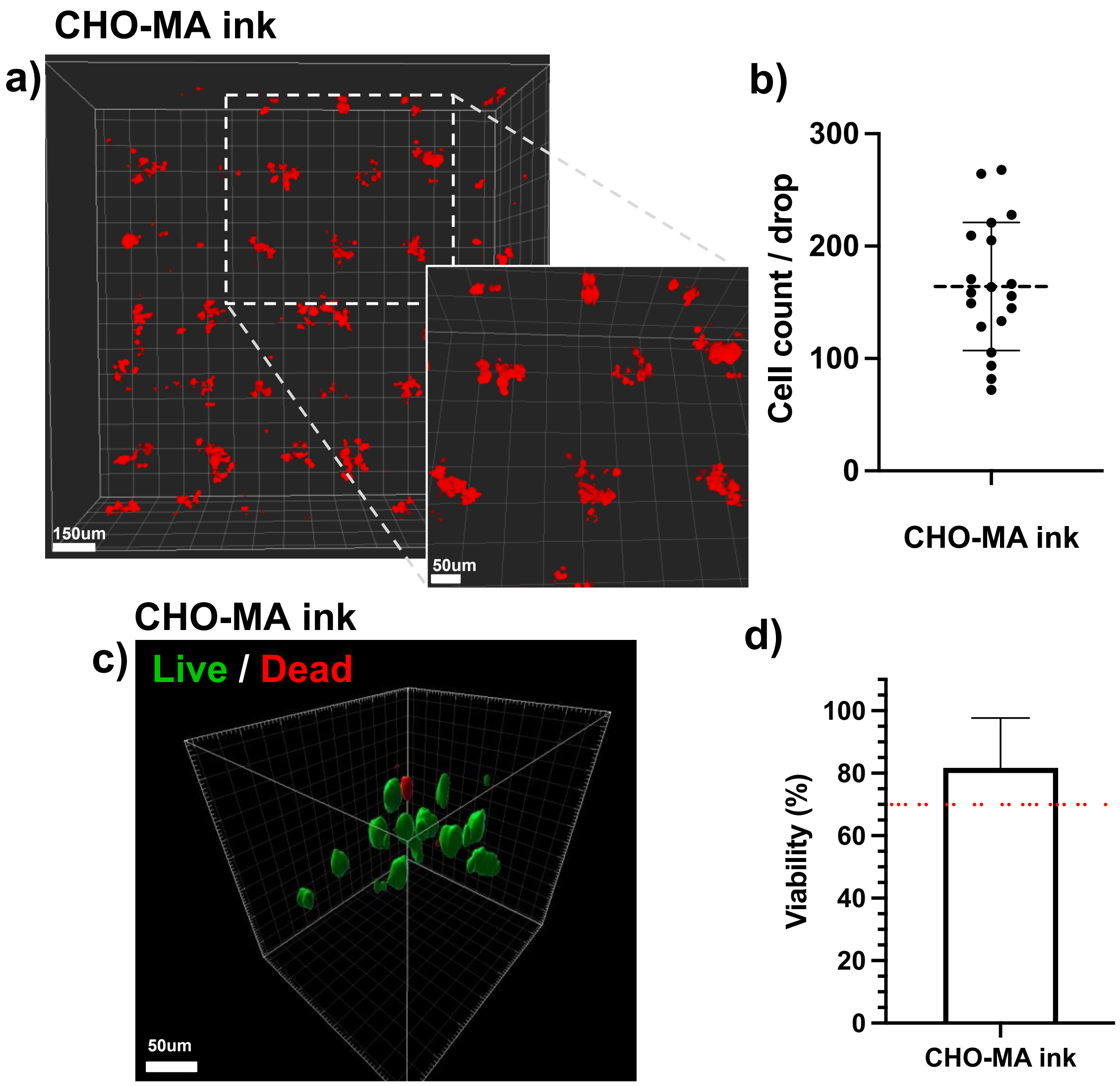

Figure S7- a) Confocal imaging of laser assisted bioprinted RFP+ human umbilical vein endothelial cells (HUVECs, red) with methacrylated chorionic membrane (0.6% w/v, CHO-MA ink), one day post bioprinting. Biomaterial ink contained 0.15 % w/v of photoinitiator LAP. Bioprinting considered the use of the Modulab (Alphanov, France), HUVECs were printed at 70 millions/mL in cell culture medium. Laser-assisted settings considered a 2 nanosecond impulsion, at 25 µJ per pulse using a far red (1064 nm) nanosecond laser. Cell spots were created every 250 µm and projected between two CHO-MA ink layers, followed by a 1 min 365 nm UV photopolymerization, and cultured in EGM-2 MV medium (Lonza, France). b) Cell number by droplet was quantified using image analysis (Imaris) and plotted as cell count per drop (Aver±SD). c) Confocal imaging of Live-Dead assay over a representative laser-assisted bioprinted spot of human umbilical vein endothelial cells (HUVECs) bioprinted as described above and at 24 hrs post printing (scale=50 µm, Live cells=green, dead cells= red). d) Viability percentage (%) of laser-assisted bioprinted HUVECs at 24 hrs post printing. Briefly, Live-dead assay was performed following staining with calcein-AM ("Live", 1 µL/mL, Invitrogen) and ethidium

homodimer (“Dead”, 4 µL/mL, Invitrogen) for 20 min. Cell were analyzed by confocal microscopy and analyzed using ImageJ for live (positive for calcein) and dead. “Live” cell percentage was determined (n = 8 replicates, Aver±SD; please note that viability average values higher than 70% viability).

Table S1 A- Top core matrisome proteins identified and categorized, % of coverage, number of unique peptides and abundances for amnion and chorion.

Protein subtype	Protein name	Gene symbol	Coverage [%]	Unique peptides	Abundances (Grouped): Amnion	Abundances (Grouped): Chorion
Collagen subtypes	Collagen alpha-1(I)	COL1A1	43	43	6.09E+09	5.82E+09
	Collagen alpha-2(I)	COL1A2	40	36	4.96E+09	5.85E+09
	Collagen alpha-1(II)	COL2A1	7	4	1.85E+08	1.89E+08
	Collagen alpha-1(III)	COL3A1	24	23	1.99E+09	1.16E+09
	Collagen alpha-1(IV)	COL4A1	2	2	1.17E+06	8.91E+06
	Collagen alpha-2(IV)	COL4A2	10	10	6.12E+06	5.71E+07
	Collagen alpha-1(V)	COL5A1	8	11	2.98E+08	3.53E+08
	Collagen alpha-2(V)	COL5A2	16	5	3.17E+07	4.62E+07
	Collagen alpha-1(VI)	COL6A1	4	2	1.96E+06	3.40E+06
	Collagen alpha-2(VI)	COL6A2	8	6	7.85E+06	3.85E+07
	Collagen alpha-3(VI)	COL6A3	6	18	1.49E+08	1.88E+08
	Collagen alpha-1(VII)	COL7A1	1	3	7.84E+04	5.47E+04
	Collagen alpha-1(XI)	COL11A1	5	5	1.55E+07	2.61E+07
Other extracellular matrix proteins	Dermatopontin	DPT	16	3	4.20E+04	4.20E+04
	EMILIN-2	EMILIN2	2	2	4.73E+04	9.75E+04
	Fibrillin-1	FBN1	27	51	1.86E+07	1.18E+08
	Fibrillin-2	FBN2	5	9	9.78E+05	8.83E+06
	Fibronectin	FN1	16	27	4.81E+06	5.72E+07
	Heparan sulfate proteoglycan core protein	HSPG2	1	2	2.53E+04	3.86E+05
	Laminin subunit alpha-2	LAMA2	1	3	4.81E+04	3.01E+05
	Laminin subunit alpha-5	LAMA5	1	3	1.26E+05	5.35E+05
	Laminin subunit beta-1	LAMB1	8	5	5.78E+05	6.21E+05
	Laminin subunit beta-2	LAMB2	14	13	3.34E+05	5.86E+06
	Laminin subunit gamma-1	LAMC1	9	9	1.46E+05	3.47E+06
	Tenascin-X	TNXB	5	13	1.36E+06	2.56E+06
	Thrombospondin-1	THBS1	2	2	4.25E+04	4.36E+04
Structural proteins	Agrin	AGRN	2	3	3.33E+04	6.37E+04
	Alpha-globin	HBA1	19	3	6.62E+04	4.85E+05
	Annexin	ANXA3	7	2	5.26E+06	6.09E+06
	Annexin A1	ANXA1	38	10	2.21E+05	7.21E+05
	Annexin A2	ANXA2	57	17	4.53E+05	3.93E+06
	Annexin A5	ANXA5	25	6	1.71E+05	3.54E+05
	Caveolin	CAV1	24	3	6.33E+04	4.68E+05
	Derlin-1	DERL1	4	2	3.11E+04	1.45E+04
	Desmoglein-1	DSG1	3	2	3.46E+04	1.63E+04
	ER lumen protein-retaining receptor 1	KDELR1	20	2	3.30E+04	1.96E+05
	Fibrinogen alpha chain	FGA	1	2	4.90E+04	9.60E+05
	Fibrinogen beta chain	FGB	7	3	7.30E+04	5.75E+05
	Filamin-B	FLNB	5	2	3.92E+04	3.73E+04
	Gap junction alpha-1 protein	GJA1	19	5	1.02E+05	1.03E+06
	Glia-derived nexin	SERPINE2	24	9	1.93E+05	8.60E+05
	Hemoglobin subunit beta	HBB	63	4	8.11E+04	5.12E+05
	Histocompatibility 13 isoform 2	HM13	10	4	9.62E+04	2.12E+06
	Integrin beta	ITGB1	6	4	2.66E+05	4.73E+05
	Lysosome-associated membrane glycoprotein 1	LAMP1	10	4	5.46E+05	4.25E+05
	Lysosome-associated membrane glycoprotein 2	LAMP2	5	2	4.42E+04	1.86E+05
	Pituitary tumor-transforming gene 1	PTTG1IP	14	2	2.40E+04	5.11E+05
	Plectin	PLEC	1	6	2.28E+05	3.67E+06
	Prelamin-A/C	LMNA	6	4	6.44E+04	6.49E+04
	Prosaposin	PSAP	5	3	3.29E+04	4.19E+04
	Sorting and assembly machinery component 50 homolog	SAMM50	5	2	3.32E+04	3.52E+04
	Surfeit locus protein 4	SURF4	14	3	7.09E+04	8.72E+05
	Synaptophysin-like protein 1	SYPL1	10	2	6.96E+04	7.15E+05
	Transformer-2 protein homolog beta	TRA2B	10	2	3.12E+04	2.18E+04
	Translocating chain-associated membrane protein 1	TRAM1	6	2	4.24E+04	3.36E+05
	Transmembrane protein 43	TMEM43	5	2	9.41E+04	2.21E+05
	Tropomyosin 1 (Alpha), isoform CRA_o	TPM1	16	3	6.46E+04	1.11E+05
	Tropomyosin beta chain	TPM2	12	2	4.63E+04	5.92E+04
	Tubulin alpha chain	TUBA1C	5	2	5.15E+04	1.12E+05
	Voltage-dependent anion-selective channel protein 1	VDAC1	80	15	1.49E+06	1.20E+07
	Voltage-dependent anion-selective channel protein 2	VDAC2	55	11	3.42E+05	1.71E+07
	Voltage-dependent anion-selective channel protein 3	VDAC3	19	3	3.21E+04	5.01E+05

Table S1 B- Top core matrisome proteins identified and categorized, % of coverage, number of unique peptides and abundances for amnion and chorion.

Protein subtype	Protein name	Gene symbol	Coverage [%]	Unique peptides	Abundances (Grouped): Amnion	Abundances (Grouped): Chorion
Transport protein	Antigen peptide transporter 1	TAP1	4	2	4.89E+04	1.99E+05
	Apolipoprotein B-100	APOB	1	5	5.70E+04	4.56E+04
	B-cell receptor-associated protein	BCAP31	13	2	2.45E+04	7.27E+04
	Choline transporter-like protein 1	SLC44A1	3	2	4.62E+04	8.29E+04
	Choline transporter-like protein 2	SLC44A2	12	8	1.55E+05	1.43E+06
	Dickkopf-related protein 1	DKK1	14	4	6.95E+04	5.51E+06
	Leukocyte surface antigen CD47	CD47	9	3	6.48E+04	2.38E+05
	Lipocalin-1	LCN1	13	2	1.44E+05	3.84E+04
	P-type Ca(2+) transporter	ATP2A2	5	4	4.31E+04	3.18E+05
	Phosphate carrier protein, mitochondrial	SLC25A3	5	2	5.37E+04	1.07E+05
	Plasma membrane calcium-transporting ATPase 4	ATP2B4	1	2	5.13E+04	2.42E+05
	Solute carrier family 2, facilitated glucose transporter member 1	SLC2A1	12	3	9.77E+04	1.79E+06
	Solute carrier family 2, facilitated glucose transporter member 3	SLC2A3	4	2	3.69E+04	5.00E+05
Enzymatic protein	Acidic mammalian chitinase	CHIA	9	5	4.11E+08	5.48E+07
	Dolichyl-diphosphooligosaccharide--protein glycosyltransferase	DAD1	48	5	9.78E+04	1.12E+06
	Dolichyl-diphosphooligosaccharide--protein glycosyltransferase	STT3A	6	3	7.29E+04	5.34E+05
	Glyceraldehyde-3-phosphate dehydrogenase	GAPDH	8	2	1.11E+05	2.32E+05
	Metalloproteinase inhibitor 3	TIMP3	28	6	1.52E+05	1.22E+06
	Microsomal glutathione S-transferase 3	MGST3	18	3	9.82E+04	1.44E+06
	Prostaglandin E synthase	PTGES	30	3	4.78E+04	1.17E+05
	Protein-glutamine gamma-glutamyltransferase 2	TGM2	9	7	1.73E+05	1.14E+06
	Serine protease 1	PRSS1	11	2	2.18E+07	9.28E+06
	Trypsin-3	PRSS3	10	2	9.02E+04	3.87E+05
Contractile protein	Myosin-10	MYH10	2	3	1.00E+04	1.47E+05
Immune System related protein	Alpha-1-antichymotrypsin	SERPINA3	29	10	1.83E+05	2.25E+05
	Antileukoproteinase	SLPI	53	9	6.51E+05	6.79E+06
	C4b-binding protein alpha chain	C4BPA	4	2	5.03E+04	6.07E+04
	Complement component C8 beta chain	C8B	6	3	3.26E+04	1.28E+05
	Complement decay-accelerating factor	CD55	16	4	4.81E+05	8.16E+05
	Immunoglobulin heavy constant gamma 4	IGHG4	7	2	4.83E+04	4.83E+04
	Myeloid-associated differentiation marker	MYADM	12	3	1.01E+05	5.52E+06
	Neutrophil defensin 1	DEFA1B	26	3	6.40E+04	1.27E+07
	Norrin	NDP	18	2	3.37E+04	5.11E+05
	Plasma protease C1 inhibitor	SERPING1	12	5	5.64E+04	5.56E+04
	Protein kish-A	TMEM167A	25	2	3.94E+04	2.88E+05
Intermediate filament protein	Keratin, type I cytoskeletal 10	KRT10	59	26	6.01E+07	6.20E+07
	Keratin, type I cytoskeletal 14	KRT14	29	5	1.54E+06	5.29E+05
	Keratin, type I cytoskeletal 17	KRT17	14	2	2.73E+04	8.57E+03
	Keratin, type I cytoskeletal 19	KRT19	16	3	6.83E+04	8.34E+05
	Keratin, type I cytoskeletal 9	KRT9	73	31	4.81E+07	4.31E+07
	Keratin, type II cytoskeletal 1	KRT1	63	2	1.30E+07	1.20E+07
	Keratin, type II cytoskeletal 1b	KRT77	8	2	7.31E+06	6.27E+06
	Keratin, type II cytoskeletal 2 epidermal	KRT2	68	27	2.37E+07	3.36E+07
	Keratin, type II cytoskeletal 5	KRT5	26	6	2.25E+06	1.89E+06
	Keratin, type II cytoskeletal 6A	KRT6A	24	3	4.80E+05	3.28E+05
Proteoglycan	Bone marrow proteoglycan	PRG2	40	7	2.51E+05	1.94E+07
